# Management of *BRAF* Gene Alterations in Metastatic Colorectal Cancer: From Current Therapeutic Strategies to Future Perspectives

**DOI:** 10.3389/fonc.2021.602194

**Published:** 2021-03-25

**Authors:** Hiroyuki Takeda, Yu Sunakawa

**Affiliations:** Department of Clinical Oncology, St Marianna University School of Medicine, Kawasaki, Japan

**Keywords:** colorectal cancer, *BRAF* mutation, *BRAF* V600E, *BRAF* non-V600E, microsatellite instability (MSI)

## Abstract

*BRAF* mutations constitute an important poor prognostic factor in metastatic colorectal cancer (mCRC) and the development of treatments in this context is of great necessity to prolong patient survival. Although the association between *BRAF* mutations and microsatellite instability (MSI) has been known for several years, previous clinical trials have revealed that the former has a limited prognostic impact and that immune checkpoint inhibitors offer a significant survival benefit to mCRC patients with both characteristics. Furthermore, the genomic classification of *BRAF* mutations according to their molecular functions enables greater understanding of the characteristics of mCRC patients with *BRAF* mutations, with therapeutic strategies based on this classification made more ideal to improve poor prognosis through the delivery of targeted therapies. Recently, a phase III trial was conducted in previously treated mCRC patients with *BRAF* V600E–mutated tumors and revealed that the combination therapy approach of *BRAF* inhibition and anti–epidermal growth factor receptor antibody therapy with or without MEK inhibition was more efficacious than standard chemotherapy alone. This review discusses current treatment strategies and future perspectives in *BRAF*-mutated mCRC.

## Introduction

Colorectal cancer (CRC) is one of the most common cancers, with an associated mortality rate of 9.2% that makes it the second leading cause of cancer-associated deaths (1). Early-stage CRC can be curable under surgery, whereas metastatic or recurrent CRC is usually unresectable and carries a poor prognosis. In recent years, treatments targeting vascular endothelial growth factor (VEGF) receptors and epidermal growth factor receptors (EGFRs) have been reported to significantly extend the survival of metastatic CRC (mCRC). Furthermore, various biomarker studies, including concerning the *RAS* gene, have been also conducted and some findings have been deemed clinically useful in practice. In particular, *BRAF* mutations have been shown collectively to be a remarkably poor prognostic factor (2, 3). BRAF is part of the RAS–RAF–MEK intracellular signaling pathway and its mutation is considered a genetic aberration that activates a signal that promotes tumor growth. BRAF is a kinase protein located downstream of EGFR, suggesting that patients with *BRAF*-mutated mCRC are less likely to benefit from anti-EGFR agents. However, a few clinical trials to date have suggested additional benefits of anti-EGFR therapy, so controversy therefore remains (4, 5). The use of fluorouracil/folinic acid, oxaliplatin, and irinotecan (FOLFOXIRI) plus bevacizumab is considered to be a promising treatment regimen to prolong survival for *BRAF* V600E–mutated mCRC. However, recent reports indicate that further investigation is required (6). This article summarizes the data of previous clinical trials in *BRAF*-mutated mCRC, discusses current treatment strategies, and offers future perspectives.

## The Roles of *BRAF* Mutation in Colorectal Cancer

**Figure 1:**
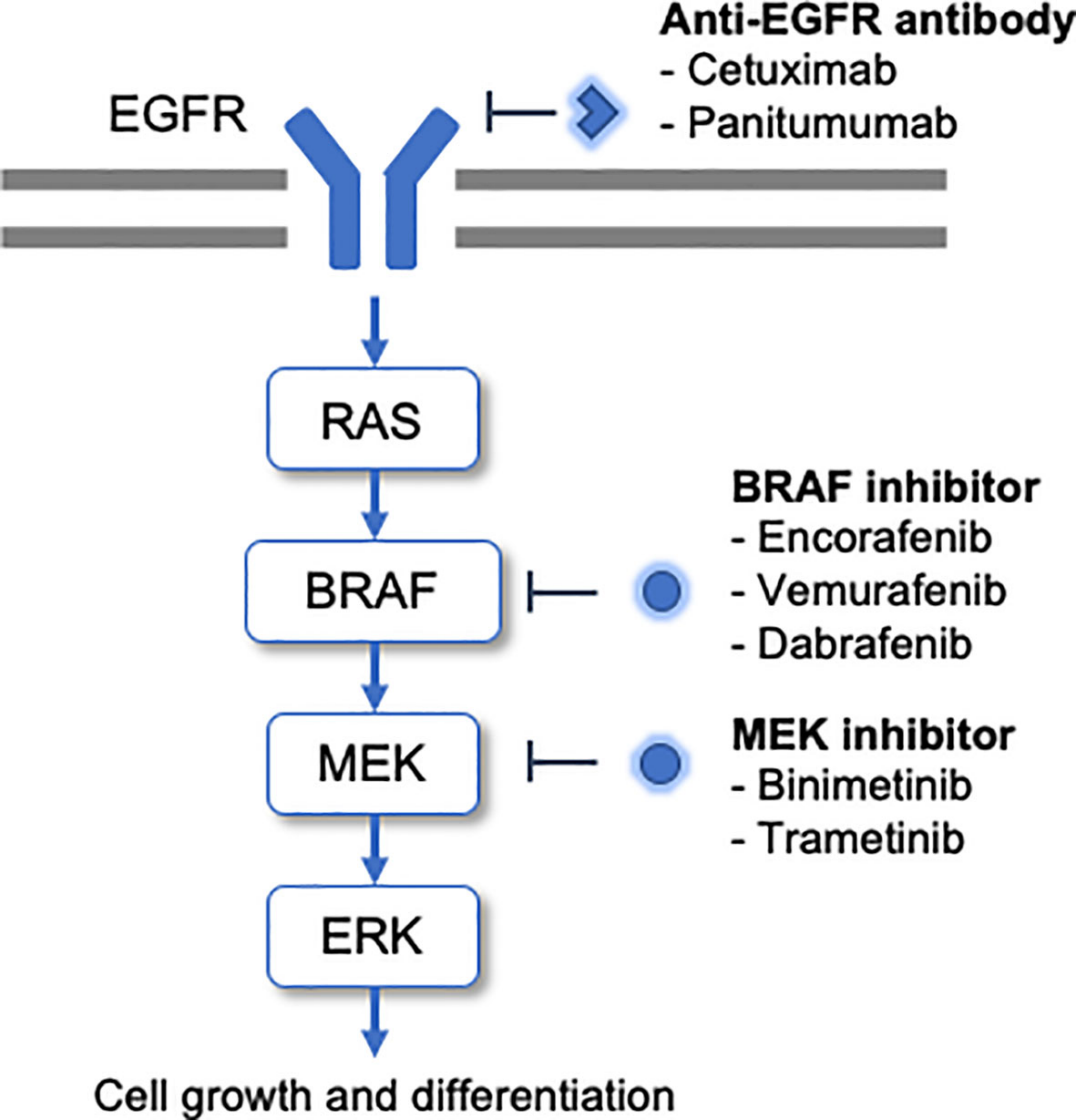
*BRAF* mutations promote the activation of this pathway and tumorigenesis. The molecular-targeted drugs developed to date for *BRAF*-mutated CRC are shown alongside the cascade.

BRAF is a serine/threonine-specific protein kinase involved in the signaling cascade of the mitogen-activated protein kinase (MAPK) pathway, which promotes cell growth and differentiation (**Figure 1**). Activated RAF proteins trigger the activation of MEK1/2 and further activate ERK. Subsequently, ERK phosphorylates transcription factors and regulates significant cellular activity (7, 8). Approximately 15% to 30% of all CRCs are thought to contribute to cancer in the serrated pathway, a multistage carcinogenic mechanism that is an alternative to the traditional adenomatous carcinoma model. Morphologically, serrated lesions can be classified as hyperplastic polyps, serrated adenomas/polyps, and classic serrated adenomas, respectively. These lesions exhibit a high incidence of *BRAF* mutations and also present the CpG island methylator phenotype (CIMP), which causes methylation of mismatch repair genes and can progress to microsatellite instability (MSI) (9).

*BRAF* mutations are found in 10% to 15% of CRCs, including early-stage cancers (10). In addition, the frequency of *BRAF* mutations in mCRC has been reported to be 8% to 10% according to prior retrospective analyses of several clinical trials (3, 11, 12). *BRAF* mutations are a key poor prognostic factor in this population, affecting the progression-free survival (PFS) and overall survival (OS) alike (13–15). The prognostic impact of *BRAF* mutations is generally similar in between CRCs and melanomas, whereas in lung cancers, there are conflicting results among studies, possibly due to their low frequency (16, 17). Patients with *BRAF*-mutated CRC are more likely to be female, have tumors on the right side, exhibit more peritoneal metastases, and show more mucinous histology in comparison with patients with wild-type CRC (18–20). Most *BRAF* variants present with mutations in *BRAF* V600E. Most recently, it has become recognized that clinical features differ depending upon the site of the mutation.

## Alterations in the *BRAF* Gene in Patients With mCRC

Most *BRAF*-mutated mCRC have mutations in V600E, but 2% to 3% show other mutations (19, 21). Non-V600 *BRAF*-mutant mCRC was reported to involve different clinical characteristics relative to V600 *BRAF*-mutant mCRC; typically, patients with non-V600 *BRAF*-mutant mCRC are slightly younger and are less female, with low-grade tumors and with primary tumors located on the left side more often (19). In terms of prognosis, the median OS was longer in patients with non-V600 *BRAF*-mutant mCRC as compared with among both V600 *BRAF*-mutant mCRC and *BRAF* wild-type mCRC patients. In patients with NSCLC, the prognostic impact of non-V600 mutations is not consistent across reports (22, 23), while in patients with melanoma, there was no significant survival difference between non-V600 mutations, V600 *BRAF*-mutation, and *BRAF* wild-type (24).

Based on recent analysis, *BRAF* mutations can be classified into three groups according to their function (25, 26). Class 1, encompassing V600 mutations, is marked by high kinase activity and exhibit MEK/ERK signaling activation as RAS-independent monomers. Class 2 mutations involve intermediate kinase activity and exhibit RAS-independent activation of MEK/ERK signaling in a dimer with BRAF. Meanwhile, class 3 mutations exhibit reduced kinase activity and are dependent on RAS to activate signaling (27). Notably, class 3 mutations are more likely to be associated with long-term survival relative to both class 1 and class 2 mutations (28). Moreover, it has been reported that patients with *BRAF* mutations may respond differently to treatments depending on the mutation class. *BRAF* mutations of classes 2 and 3 are not responsive to RAF inhibitors, whereas class 3 mutants are RAS-dependent and potentially effective for EGFR inhibition (27). Confirming this, a large series analysis of non-V600 *BRAF*-mutant CRC patients receiving anti-EGFR therapy revealed that patients with class 3 *BRAF* mutations responded to anti-EGFR therapy, while those with class 2 *BRAF* mutations did not responded (29). On the contrary, two studies have reported that non-V600 *BRAF* mutations are unable to be mitigated with anti-EGFR therapy (30, 31). Owing to the rarity of non-V600 *BRAF* mutations in this population, the efficacy of anti-EGFR therapy and the optimal regimen for non-V600 *BRAF*-mutant CRC remains unclear. A multicenter phase II trial of the combination of the MEK inhibitor binimetinib, the BRAF inhibitor encorafenib, and the anti-EGFR antibody drug cetuximab is currently ongoing, assessing patients with non-V600E *BRAF*-mutant mCRC who have not received any previous anti-EGFR antibody drugs. This study will also ultimately test the same regimen in class 3 patients with prior anti-EGFR antibody exposure (32).

Acquired *BRAF* alterations also may occur. *BRAF* amplification has been reported in patients with *BRAF*-mutated mCRC who have developed resistance to combination RAF/MEK inhibition. This alteration results in the onset of resistance to the RAF/EGFR or RAF/MEK combination through sustained MAPK pathway activity (33). Thus, therapeutic strategies to address the *BRAF* mutation class and resistance through *BRAF* amplification are also attracting attention regarding non-*BRAF* V600E.

## The Effect of Conventional Chemotherapy on *BRAF*-Mutated mCRC

Clinical trials assessing the impact of conventional chemotherapy, including both as first-line and second-line treatment, are shown in **Table 1**. The efficacy of anti-EGFR therapy for *BRAF*-mutated mCRC is controversial. In the pooled analysis of CRYSTAL and OPUS, two randomized clinical trials examining the effects of adding cetuximab to doublet chemotherapy, anti-EGFR treatment displayed numerical benefits in the objective response rate (ORR), PFS, and OS for *BRAF*-mutated mCRC, although no significant difference was recorded. Anti-EGFR plus chemotherapy presented an ORR of 21.9%, median PFS of 7.1 months, and median OS of 14.1 months; in contrast, chemotherapy alone demonstrated an ORR of 13.2%, median PFS of 3.7 months, and median OS of 9.9 months (3).

**Table 1:** Efficacy of conventional chemotherapies for *BRAF*-mutated metastatic colorectal cancer.

Trial	Author	Design	Treatment	Line		N	ORR (%)	mPFS (months)	mOS (months)
**FIRST LINE**									
**DOUBLET vs. DOUBLET**									
NCT0265850 (CALGB/SWOG 80405)	Innocenti et al.	Retrospective analysis	FOLFOX/FOLFIRI + Bev	1		41	NA	7.6	15.0
–	–	–	FOLFOX/FOLFIRI + Cet	–		33	NA	6.2	11.7
–	–	–	FOLFOX/FOLFIRI + Bev + Cet	–		26	NA	8.6	16.4
NCT00433927 (FIRE-3)	Stintzing et al.	Retrospective analysis	FOLFIRI + Bev	1		23	40	6.6	13.7
–	–	–	FOLFIRI + Cet	–		25	52	6.6	12.3
**TRIPLET**									
NCT01163396	Masi et al.	Retrospective analysis	FOLFOXIRI + Bev	1		10	90	12.8	23.8
NCT01437618	Loupakis et al.	Phase II	FOLFOXIRI + Bev	1		15	60	9.2	24.1
NCT01328171 (VOLFI)	Modest et al.	Subgroup analysis in phase II	mFOLFOXIRI + Pani	1		7	86	6.5	NA
		–	FOLFOXIRI	–		9	22	6.1	NA
**TRIPLET vs. DOUBLET**									
NCT00719797 (TRIBE)	Cremolini et al.	Subgroup analysis in phase III	FOLFOXIRI + Bev	1		16	56	7.5	19.0
		–	FOLFIRI + Bev	–		12	42	5.5	10.7
NCT01321957(CHARTA)	Schmoll et al.	Subgroup analysis in phase II	FOLFOXIRI + Bev	1		8	83	10.1	NA
		–	FOLFOX + Bev	–		5	80	7.8	NA
NCT01765582 (STEAM)	Hurwitz et al.	Subgroup analysis in phase II	FOLFOXIRI + Bev	1		4	50	7.1	NA
		–	sFOLFOXIRI + Bev (FOLFOX + Bev alternating with FOLFIRI + Bev)	–		5	80	7.4	NA
		–	FOLFOX + Bev	–		4	75	12.4	NA
NCT02339116(TRIBE2)	Cremolini et al.	Subgroup analysis in phase III	Upfront FOLFOXIRI + Bev followed by the same regimen	1		33	NA	1st HR 1.02 (0.61–1.71) 2nd HR 1.23 (0.72–2.09)	HR 1.35 (0.79–2.30)
			mFOLFOX6 + Bev followed by FOLFIRI + Bev	–		33	NA	–	–
**SECOND LINE**									
NCT01754272 (VELOUR)	Wirapati et al.	Subgroup analysis in phase III	FOLFIRI + aflibercept		2	16	NA	5.5	10.3
		–	FOLFIRI + placebo		–	20	NA	2.2	5.5
NCT01183780 (RAISE)	Yoshino et al.	Subgroup analysis in phase III	FOLFIRI + ramcirumab		2	20	NA	5.7	9.0
		–	FOLFIRI + placebo		–	21	NA	2.7	4.2

Two meta-analyses to date have evaluated the influence of anti-EGFR therapy in *BRAF*-mutated mCRC. Although these reports suggested a trend existed toward better hazard ratios (HRs) concerning PFS and OS in the context of EGFR therapy, it did not show a statistically significant difference (4, 5). Recently, the VOLFI trial (AIO KRK0109), a randomized phase II trial investigating the addition of panitumumab to triplet chemotherapy with FOLFOXIRI, reported results for the subgroup of patients with *BRAF*-mutated mCRC. The ORR of the FOLFOXIRI plus panitumumab arm was 86% and was higher as compared with that of 22% in the FOLFOXIRI-alone arm. In contrast with the effect of ORR, the prolongation of PFS was modest, as PFS was 6.5 and 6.1 months for the FOLFOXIRI plus panitumumab and FOLFOXIRI-alone arms, respectively (34). These results support that anti-EGFR therapy may show some efficacy for the treatment of *BRAF*-mutated mCRC, although the magnitude of efficacy is less than that of *RAS/BRAF* wild-type mCRC. Further accumulation of evidence and molecular analyses may be necessary to determine whether anti-EGFR therapy truly offers a degree of efficacy that is beneficial enough.

The efficacy of anti-VEGF therapy for *BRAF*-mutated mCRC has been also discussed. A biomarker analysis of a phase III study—the first report of the efficacy of bevacizumab in previously untreated patients with mCRC—included 10 patients with *BRAF* mutations. The median survival was 16 months among seven patients in the bevacizumab group and eight months for three patients in the placebo group, with an HR of 0.11 [95% confidence interval (CI): 0.01–1.06] (35). However, as the number of patients included was very small, it is not clear whether bevacizumab had the same effect in *BRAF*-mutated mCRC that it does in other types of mCRC. As second-line treatment, the efficacy of anti-VEGF targets against *BRAF*-mutated tumors has been reported in a relatively large number of patients. Among 41 patients with *BRAF* mutation, the effect of ramucirumab, a monoclonal antibody drug targeting VEGFR2, relative to that of placebo was demonstrated in a subgroup analysis of the RAISE trial, with HRs of 0.54 (95% CI: 025–1.13) and 0.55 (95% CI: 0.28–1.08) for PFS and OS, respectively (36). A subgroup analysis of *BRAF* mutants in the VELOUR trial, a phase III study that proved the efficacy of aflibercept, also suggested that the efficacy of anti-VEGF therapy was preserved, with an HR of 0.59 (95% CI: 0.22–1.58) for PFS and that of 0.42 (95% CI: 0.16–1.09) for OS (37).

Meanwhile, in the CALGB/SWOG80405 and FIRE-3 trials, where bevacizumab was compared with cetuximab as first-line treatment, retrospective analyses were conducted for *BRAF*-mutated mCRC (14, 38). For *BRAF*-mutated mCRC, the median PFS in the bevacizumab group and the cetuximab group were similar in these studies; the median PFS outcomes in the bevacizumab group were 7.6 months and 6.6 months, and those in the cetuximab group were 6.2 months and 6.6 months, respectively. Further, the median OS tended to be slightly longer for bevacizumab, garnering median OS outcomes of 15.0 and 13.7 months in the bevacizumab group and 11.7 and 12.3 months in the cetuximab group. Ultimately, however, it remains uncertain which therapeutic option (bevacizumab or anti-EGFR antibody medication) as first-line treatment is more favorable for addressing *BRAF*-mutated mCRC.

FOLFOXIRI plus bevacizumab is considered to be one of the leading regimens against *BRAF-*mutated mCRC based on evidence from both retrospective and prospective trials involving small numbers of patients as well as the results of a subgroup analysis of the TRIBE trial. A retrospective analysis of a phase II study of FOLFOXIRI plus bevacizumab indicated that the response rate was 90%, the PFS was 12.8 months, and the OS was 23.8 months among 10 patients with *BRAF*-mutated mCRC (39). Elsewhere, a prospective study was conducted involving 15 patients with *BRAF*-mutated mCRC and FOLFOXIRI plus bevacizumab ensured a response rate of 60%, a PFS of 9.2 months, and an OS of 24.1 months, with these results being comparable to those of the previous report (40). In the TRIBE trial, FOLFOXIRI plus bevacizumab (triplet arm) versus FOLIRI plus bevacizumab (doublet arm) in the context of mCRC were compared. A subgroup analysis of *BRAF*-mutated tumors was also completed to confirm the previously described efficacy of the triplet regimen for *BRAF*-mutated mCRC. The results showed that the response rate was 56% in the triplet group (n = 16 patients) and 42% in the doublet group (n = 12 patients), PFS outcomes of 7.5 months versus 5.5 months and OS outcomes of 19.0 months versus 10.7 months, indicating that the triplet group was more favorable for *BRAF*-mutated tumors (6). In the subgroup analysis of the STEAM and CHART trials, the HRs for the median PFS were 0.8 (95% CI: 0.2–3.0) and 0.72 (95% CI: 0.25–2.07), respectively, which were not significantly different but suggested that the triplet regimen was a slightly better option (41, 42). A pooled analysis including these studies reported an HR of 0.65 (95% CI: 0.33–1.26) for the triplet group, indicating no significant difference, but suggesting that the triplet regimen was again preferable (41).

However, recently, a subgroup analysis of the TRIBE2 trial presented a different trend as compared with in previous studies (43). A total of 33 patients with *BRAF*-mutated mCRC were present in both the experimental and control groups, with an HR for the PFS of 1.02 (95% CI: 0.61–1.71), while the HR for PFS2, defined as the time from randomization to disease progression on any treatment given after the first instance of disease progression, was 1.23 (95% CI: 0.72–2.09). Furthermore, the OS presented an HR of 1.35 (95% CI: 0.79–2.30), which indicates considerable uncertainty regarding whether the triplet therapy approach is beneficial for *BRAF*-mutated mCRC. A meta-analysis of these five studies using individual patient data reported an HR of 1.11 (95% CI: 0.75–1.73) for the OS of the triplet regimen versus the doublet regimen, with an uncertain level of benefit noted in patients with *BRAF*-mutated mCRC (44). In addition, using real-world data from the United States, a retrospective evaluation of triplet and doublet regimens for patients with *BRAF*-mutated mCRC was conducted and showed that 16 patients receiving the triplet regimen and 423 patients receiving the doublet regimen showed median OS outcomes of 13.8 months and 15.5 months (*p* = 0.38), respectively (45). In summary, the benefit of the triplet plus bevacizumab regimen in *BRAF*-mutated tumors is controversial and adequate prospective validation in *BRAF*-mutated tumors is becoming more crucial. Also, there is insufficient evidence of the additive effect of anti-VEGF–targeted drugs in *BRAF*-mutated mCRC, but no negative evidence is currently available; thus, anti-VEGF drugs are recommended for patients for whom anti-VEGF therapy is accessible. There is no specific regimen recommended for first-line therapy of *BRAF*-mutated tumors, as the National Comprehensive Cancer Network guidelines recommend using the same regimen as that for the *RAS* wild type tumors.

## Current Treatment Strategies for *BRAF*-Mutated mCRC

Therapeutic strategies encompassing BRAF and MEK inhibitors for melanoma and thyroid cancer with *BRAF* mutation have been successful and several BRAF and MEK inhibitors have already been approved to date by the United States Food and Drug Administration for these diseases (46). In this section, the current development of BRAF and MEK inhibitors for the management of *BRAF*-mutated mCRC will be described (**Table 2**). In contrast with in melanoma, monotherapy with BRAF inhibitors has not been quite as successful in mCRC. For example, a phase I dose-escalation study of encorafenib was conducted in 18 patients with *BRAF* V600E–mutated mCRC and none of the patients showed a confirmed response. In 12 patients, however, the best response was achieved with stable disease, with a median PFS of 4.0 months (47). Elsewhere, the effects of vemurafenib were assessed in 21 patients with *BRAF*-mutated mCRC, with only one partial response. The best response with stable disease was seen in seven patients and the median PFS was 2.1 months (48). In a basket study of vemurafenib in *BRAF* V600E–mutated cancers, 10 patients with mCRC who received vemurafenib monotherapy experienced no response. Both *in vitro* and *in vivo* studies have reported that the cause of the failure to respond to BRAF inhibition alone was the rapid reactivation of ERK *via* the feedback activation of EGFR. Given this, the combination of BRAF inhibitor and anti-EGFR antibody medications was expected to increase efficacy in mCRC and the combination of vemurafenib and cetuximab was therefore attempted in another cohort. Ultimately, however, one of the 27 patients experienced a partial response (49). Moreover, in a phase I/II trial of another anti-EGFR antibody combination, 13 patients received the combination of vemurafenib and panitumumab, and two patients responded to treatment (50).

**Table 2:** Efficacy of targeted therapies for *BRAF* V600E–mutated mCRC.

Trial	Authors	Phase	Treatment	Pretreated	N	ORR (%)	PFS (months)	mOS (months)
**Encorafenib-based**								
**NCT01436656**	Gomez-Roca et al.	1	Encorafenib	≥ 1	18	0	4	N/A
**NCT01719380**	van Geel et al.	1b	Encorafenib + cetuximab	≥ 1	26	19	3.7	N/A
**-**		–	Encorafenib + cetuximab + alpelisib	–	28	18	4.2	N/A
**NCT01719380**	Tabernero et al.	2	Encorafenib + cetuximab	≥ 1	50	22	4.2	Not reached
**-**		–	Encorafenib + cetuximab + alpelisib	–	52	27	5.4	15.2
**BEACON CRC (NCT02928224)**	Kopetz et al.	3	Encorafenib + binimetinib + cetuximab	1–2	224	26	4.3	9.0
**-**		–	Encorafenib + cetuximab	–	220	20	4.2	8.4
**-**		–	Irinotecan/FOLFIRI + cetuximab	–	221	4	1.5	5.4
**Vemurafenib-based**								
**NCT00405587**	Kopetz et al.	2	Vemurafenib	≥ 1	21	5	2.1	7.7
**NCT01524978**	Hyman et al.	2	Vemurafenib	≥ 1	10	0	4.5	9.3
**-**	–	–	Vemurafenib + cetuximab	–	27	4	3.7	7.1
**NCT01791309**	Yaeger et al	1/2	Vemurafenib + panitumumab	Any	15	13	3.2	7.6
**NCT01787500**	Hong et al.	1b	Vemurafenib + cetuximab + irinotcan	Any	17	35	7.7	NA
**SWOG1406 (NCT02164916)**	Kopetz et al.	2	Vemurafenib + cetuximab + irinotcan	1–2	49	16	4.4	9.6
**-**	–	2	Cetuximab + irinotecan	–	50	4	2.0	5.9
**Dabrafenib-based**								
**NCT01072175**	Corcoran et al.	1/2	Dabrafenib + trametinib	Any	43	12	3.5	N/A
**NCT01750918**	Corcoran et al.	1/2	Dabrafenib + panitumumab	Any	20	10	3.5	13.2
**-**		–	Trametinib + panitumumab	–	31	0	2.6	8.2
		–	Dabrafenib + trametinib + panitumumab	–	91	21	4.2	9.1

Other BRAF inhibitors were also tested in combination with anti-EGFR antibody drugs. In a phase Ib study of the combination of encorafenib and cetuximab in patients with *BRAF*-mutated mCRC, five of 26 (18%) patients showed a response. The trial also included the PI3K inhibitor alpelisib in combination with encorafenib and cetuximab, with responses observed in five of 28 (18%) patients; for these two study populations, the median PFS outcomes were 3.7 months and 4.2 months, respectively (51). Subsequently, a phase II part of the same study enrolled 50 patients in the encorafenib plus cetuximab arm and 52 patients in the encorafenib plus cetuximab plus alpelisib arm, respectively, and reported response rates of 22% and 27% and median PFS outcomes of 4.2 months and 5.4 months, respectively (52). Vemurafenib has also been assessed in combination with irinotecan, a cytotoxic anticancer agent: a phase I trial of vemurafenib plus cetuximab with or without irinotecan in *BRAF*-mutated mCRC showed promising outcomes, with a response rate of 35% and a median PFS of 7.7 months (53). In the subsequent phase II study, however, the results were not as expected, with a response rate of just 16% and a median PFS of 4.4 months (54).

As the inhibition of BRAF leads to the activation of MEK-dependent signaling, simultaneously inhibiting MEK while administering a BRAF inhibitor is expected to result in a better therapeutic response. Henceforth, further studies that have attempted to inhibit both BRAF and MEK will be described. In a phase I/II study of the MEK inhibitor trametinib in combination with dabrafenib, five of 43 patients (12%) with *BRAF*-mutated mCRC achieved a partial response (55). MEK inhibitors were further studied in combination with anti-EGFR therapy: a phase I/II study of dabrafenib and trametinib plus panitumumab in comparison with dabrafenib plus panitumumab or trametinib plus panitumumab, achieved a response rate of 21% and a median PFS of 4.2 months with the triplet regimen (56). Recently, a large phase III (BEACON CRC) trial demonstrated that encorafenib plus cetuximab with or without binimetinib was superior in terms of the response rate, PFS, and OS compared to FOLFIRI/irinotecan plus cetuximab. As a result of this study, the Food and Drug Administration approved encorafenib plus cetuximab for the treatment of *BRAF* V600E–mutated mCRC (57). The updated National Comprehensive Cancer Network guidelines also recommend combining encorafenib and cetuximab for patients with *BRAF* V600E–mutated mCRC who previously progressed on first-line therapy.

## Treatment for *BRAF*-Mutated mCRC With Significant MSI (MSI-High)

In previous research, *BRAF*-mutated mCRC showed a higher percentage of MSI than *BRAF* wild-type mCRC (12.6% vs. 3%) (12). A pooled analysis of four phase III studies in mCRC reported significantly worse PFS and OS outcomes for *BRAF*-mutant mCRC in comparison with *BRAF* wild-type mCRC with microsatellite-stable tumors, but no significant difference was apparent between *BRAF-*mutant and *BRAF* wild-type mCRC with MSI-high tumors (13). Therefore, MSI may have a stronger impact on prognosis as compared with *BRAF* mutation. Several clinical trials testing the effects of anti–programmed cell death protein 1 (PD-1) antibody in MSI-high/mismatch repair-deficient mCRC included patients with *BRAF* mutations. These results showed that *BRAF* mutation was not a predictor for the efficacy of anti–PD-1 antibody in MSI-high mCRC. In the KEYNOTE-164 phase II trial of pembrolizumab in previously treated MSI-high mCRC, there were nine patients included with *BRAF* V600E mutations in cohort A (who received two or more lines of treatment) and five patients with *BRAF* V600E mutations in cohort B (who received one or more lines of treatment) and the response rates were 55% and 20%, respectively. The response rates for *BRAF*-mutant and *BRAF* wild-type mCRC were 42% and 38%, respectively, and seemed to be independent of the *BRAF* mutation status (58). In the recently reported phase III KEYNOTE-177 trial of pembrolizumab versus chemotherapy in MSI-high mCRC, a subgroup analysis of patients with *BRAF* V600E mutations showed an HR of 0.48 (95% CI: 0.27–0.86) for PFS, similar to that for *BRAF* wild-type mCRC (HR: 0.50, 95% CI 0.31–0.80) (59).

The phase II trial of nivolumab or the combination of nivolumab plus ipilimumab in MSI-high mCRC, CheckMate 142, also included patients with *BRAF*-mutated mCRC and reported a relatively high response rate. In this trial, patients with *BRAF*-mutated mCRC were grouped into three arms; patients who received two or more lines of treatment were assigned to the nivolumab group (n = 25 patients) and patients who received one or more lines of treatment (n = 29 patients) or no prior treatment (n = 17 patients) were assigned to the nivolumab plus ipilimumab group. The response rates reported for these patients were 25%, 55%, and 76%, respectively, and were comparable to those for *BRAF* wild-type patients (41.4%, 55%, and 62%, respectively) (60–62). Therefore, *BRAF*-mutated mCRC with concomitant MSI-high should be preferred for treatment strategies according to the MSI-high status.

## Future Perspectives

Several clinical trials in *BRAF* V600E–mutated mCRC are currently ongoing (**Table 3**). Of these, there are two prospective trials in the first-line treatment. The randomized phase II trial AIO-KRK-0116 (NCT04034459) compares the efficacy of FOLFOXIRI plus cetuximab to FOLFOXIRI plus bevacizumab in patients with untreated *BRAF*-mutated mCRC (63). Another one is the phase II trial, ANCHOR-CRC (NCT03693170), which will evaluate the efficacy of the combination of encorafenib, binimetinib, and cetuximab, the regimen in the BEACON CRC trial, in the first-line treatment of patients with *BRAF* V600E–mutated mCRC (64). Results of the first stage of this trial were recently presented at the virtual 22nd ESMO World Congress on Gastrointestinal Cancer; 40 patients were ultimately evaluated for efficacy, with the response rate of 50% (95% CI: 33.8–66.2), tumor shrinkage in 85%, and the median PFS of 4.9 months (95% CI: 4.4–8.1) was observed, and the study is now in the second stage (65). Although not specific to first-line therapy, a phase II trial, the IMPROVEMENT study (NCT03727763) is also ongoing to evaluate the efficacy and the safety of the combination of FOLFIRI with cetuximab and vemurafenib (66).

**Table 3:** Ongoing trials for *BRAF*-mutated mCRC.

Trial	Phase	Target	Treatment	Line	Status
NCT04034459 (FIRE-4.5/AIO-KRK-0116)	2	BRAF V600E mt	FOLFOXIRI + cetuximab vs. FOLFOXIRI + bevacizumab	1st line	Recruiting
NCT03693170 (ANCHOR CRC)	2	BRAF V600E mt	Encorafenib + binimetinib + cetuximab	1st line	Active, not recruiting
NCT03727763 (IMPROVEMENT)	2	BRAF V600E mt	FOLFIRI + cetuximab + vemurafenib	NA	Recruiting
NCT03668431	2	BRAF V600E mt	Dabrafenib + trametinib + spartalizumab (anti–PD-1)	Any line	Recruiting
NCT04017650	1/2	BRAF V600E mt with MSS	Encorafenib + cetuximab + nivolumab	2nd or 3rd line	Recruiting
NCT02906059	1b	RAS or BRAF mt	AZD1775 (Wee1 inhibitor) + irinotecan	2nd line	Recruiting
NCT02857270	1	BRAF mt	LY3214996(ERK1/2 inhibitor) ± encorafenib + cetuximab	Any line	Recruiting
NCT02278133	1/2	BRAF V600- mt with RNF43 mt and/or RSPO fusion	LGK974 (porcupine inhibitor) + encorafenib + cetuximab	After at least one standard regimen	Completed

Combination therapy with immune checkpoint inhibitors and targeted therapies is being investigated in two trials. NCT03668431 is a phase II trial investigating the combination of dabrafenib and trametinib with the PD-1 inhibitor, PDR001 (spartalizumab) in previously treated *BRAF*-mutated mCRC patients (67). A similar trial of anti–PD-1 antibody medication in combination with BRAF inhibition (NCT04017650) is ongoing in patients receiving second- and third-line treatments with encorafenib in combination with cetuximab and nivolumab (68).

Preclinical data indicate that Wee1 and ERK1/2 are located downstream of BRAF in the MAP kinase signaling cascade and are potentially important therapeutic targets, and based on this, AZD1775 (Wee1 inhibitor) and LY3214996 (ERK1/2 inhibitor) have been tested in phase I trials (69–73). In addition, activation of the Wnt pathway by *RNF43* mutation may contribute to the resistance of *BRAF*-mutated mCRC to BRAF inhibitors. Therefore, a phase I/II trial of combination therapy of Wnt pathway inhibition with WNT974 (porcupine inhibitor) and BRAF inhibition has been conducted for *BRAF*-mutant CRC with RNF43 mutation (74–76).

Although clinical trials have not progressed since then, preclinical studies have reported that CDK1 and MCL-1 are involved in apoptosis resistance in *BRAF*-mutant CRC (77, 78). Combination therapy with these inhibitors and BRAF inhibitors is expected to be developed.

The future development of treatments for *BRAF*-mutated mCRC is likely to be based on combination of BRAF inhibitors and anti-EGFR agents for untreated *BRAF*-mutated mCRC, while further investigation of combination therapy with novel agents to overcome resistance to BRAF inhibitors is expected to promoted.

## Conclusion

Advances in genetic analysis techniques and the development of therapies *via* clinical trials have significantly improved the treatment of *BRAF*-mutated mCRC to date. In *BRAF*-mutant mCRC with MSI-high, the administration of immune checkpoint inhibitors as first-line treatment led to a significant survival benefit. In contrast, standard of care in first-line treatment for microsatellite-stable *BRAF-*mutated mCRC remains chemotherapy, as the optimal regimen is still uncertain and controversial. As second-line therapy, the combination of encorafenib plus cetuximab was demonstrated to provide a survival benefit, which is one of the most promising regimens for *BRAF* V600E–mutated mCRC. However, even with this new treatment, the OS with second-line therapy is only about 10 months, which is not sufficiently longer than that in *RAS*/*BRAF* wild-type mCRC. Therefore, additional therapeutic strategies must be elucidated to control the disease in the longer term; such may require the adoption of molecularly targeted therapies in first-line treatment or the further investigation of the molecular mechanisms of resistance to *BRAF* treatment.

## Author Contributions

HT drafted the manuscript and YS revised it precisely. All authors contributed to the article and approved the submitted version.

## Conflict of Interest

YS has received consulting fees from Takeda Pharmaceutical and Daiichi Sankyo and honoraria from Takeda Pharmaceutical, Taiho Pharmaceutical, Chugai Pharma, Yakult Honsha, Sanofi, Bayer Yakuhin, Bristol-Myers Squibb Japan, Merk Biopharma, Lilly Japan, Nippon Kayaku, Kyowa Kirin.

The remaining author declares that the research was conducted in the absence of any commercial or financial relationships that could be construed as a potential conflict of interest.
